# Influence of physical activity before and during pregnancy on infant’s sleep and neurodevelopment at 1-year-old

**DOI:** 10.1038/s41598-021-87612-1

**Published:** 2021-04-14

**Authors:** Kazushige Nakahara, Takehiro Michikawa, Seiichi Morokuma, Masanobu Ogawa, Kiyoko Kato, Masafumi Sanefuji, Eiji Shibata, Mayumi Tsuji, Masayuki Shimono, Toshihiro Kawamoto, Shouichi Ohga, Koichi Kusuhara, the Japan Environment and Children’s Study Group, Michihiro Kamijima, Shin Yamazaki, Yukihiro Ohya, Reiko Kishi, Nobuo Yaegashi, Koichi Hashimoto, Chisato Mori, Shuichi Ito, Zentaro Yamagata, Hidekuni Inadera, Takeo Nakayama, Hiroyasu Iso, Masayuki Shima, Youichi Kurozawa, Narufumi Suganuma, Takahiko Katoh

**Affiliations:** 1 Department of Obstetrics and Gynecology, Graduate School of Medical Sciences, Kyushu University, Fukuoka, Japan; 2 Department of Environmental and Occupational Health, School of Medicine, Toho University, Tokyo, Japan; 3 Department of Health Sciences, Graduate School of Medical Sciences, Kyushu University, Fukuoka, 812-8582 Japan; 4 Research Center for Environmental and Developmental Medical Sciences, Kyushu University, Fukuoka, Japan; 5 Department of Pediatrics, Graduate School of Medical Sciences, Kyushu University, Fukuoka, Japan; 6 Japan Environment and Children’s Study, UOEH Subunit Center, University of Occupational and Environmental Health, Kitakyushu, Fukuoka, Japan; 7 Department of Obstetrics and Gynecology, School of Medicine, University of Occupational and Environmental Health, Kitakyushu, Fukuoka, Japan; 8 Department of Environmental Health, School of Medicine, University of Occupational and Environmental Health, Kitakyushu, Fukuoka, Japan; 9 Department of Pediatrics, School of Medicine, University of Occupational and Environmental Health, Kitakyushu, Fukuoka, Japan; 10 Graduate School of Medical Sciences Department of Occupational and Environmental Health, Nagoya City University, 1 Kawasumi, Mizuho-cho, Mizuho-ku, Nagoya, Aichi 467-8601 Japan; 11 National Institute for Environmental Studies, Tsukuba, Japan; 12 National Center for Child Health and Development, Tokyo, Japan; 13 Hokkaido University, Sapporo, Japan; 14 Tohoku University, Sendai, Japan; 15 Fukushima Medical University, Fukushima, Japan; 16 Chiba University, Chiba, Japan; 17 Yokohama City University, Yokohama, Japan; 18 University of Yamanashi, Chuo, Japan; 19 University of Toyama, Toyama, Japan; 20 Kyoto University, Kyoto, Japan; 21 Osaka University, Suita, Japan; 22 Hyogo College of Medicine, Nishinomiya, Japan; 23 Tottori University, Yonago, Japan; 24 Kochi University, Nankoku, Japan; 25 Kumamoto University, Kumamoto, Japan

**Keywords:** Health care, Medical research, Risk factors

## Abstract

The aim of this study was to investigate the association between maternal physical activity (PA) before and during pregnancy and sleep and developmental problems in 1-year-old infants. We used data from a nationwide cohort study in Japan that registered 103,062 pregnancies between 2011 and 2014. Participants were asked about their PA before and during pregnancy, and the sleep and development of their children at the age of 1 year. Maternal PA was estimated using the International Physical Activity Questionnaire and was expressed in METs per week. We defined scores below the cut-off points of the Ages and Stages Questionnaire (ASQ) as abnormal for infant development. Based on the levels of PA before or during pregnancy, the participants were divided into five groups. In mothers with higher PA levels, the risk ratio for bedtime after 22:00 or abnormal ASQ scores in their 1-years-old infants were lower. These associations were observed for PA before and during pregnancy. Higher levels of maternal PA, both before and during pregnancy, may reduce sleep and developmental problems in infants.

## Introduction

Many previous studies have reported that maternal physical activity (PA) and exercise during pregnancy are associated with decreases in various perinatal outcomes, such as gestational diabetes mellitus (GDM)^1^, hypertensive disorders of pregnancy (HDP)^2^, and risk for cesarean section^3,4^. In recent years, a positive association between PA during pregnancy and infant development has been reported. Two review articles in 2018 concluded that PA during pregnancy was associated with improved language development^5^ and total neurodevelopment^6^. To our knowledge, no published studies have reported the association between maternal PA or exercise before pregnancy and infant neurodevelopment.

Recently, the importance of preconception care has become more recognized^7–9^. For example, moderate exercise prior to conception is recommended by the American College of Obstetricians and Gynecologists^8^. However, the publication focused on the benefits of exercising for improving obesity and reducing perinatal complications, and the statement did not mention infant development. It has also been reported that exercise before pregnancy reduces GDM^10^ . GDM increases the risk of developmental disorders^11^; therefore, the level of PA or exercise during preconception may affect infant development. Children with developmental disorders, such as autism, tend to have sleeping problems, including frequent awakening, crying during the night, and short sleep time due to late bedtime^12,13^. These children also tend to experience developmental delays during early infancy^14^. To our knowledge, no published studies have reported the effects of maternal PA before or during pregnancy on infant sleep. We hypothesize that higher maternal PA before and during pregnancy may improve sleep and neurodevelopment in infants.

This study aimed to investigate the association between maternal PA before and during pregnancy, and infant sleep and developmental problems at 1-year-old using large-scale data.

## Results

The baseline characteristics of the participants, along with the available data on maternal PA before pregnancy, are shown in Table 1. The characteristics of the participants with available data during pregnancy are shown in Table S1. The median (interquartile range) levels of PA before and during pregnancy were 854 (196–2975) METs-min/week and 497 (63–1386) METs-min/week, respectively.

**Table 1:** Baseline characteristics of the study population categorized by physical activity before pregnancy.

	Physical activity before pregnancy									
	PA = 0		Quartile 1		Quartile 2		Quartile 3		Quartile 4	
	n*	%	n*	%	n*	%	n*	%	n*	%
Physical activity, median (IQR), METs・min/week	0		245 (168–385)		791 (602–987)		2163 (1617–2968)		8078 (5789–12,313)	
**Maternal characteristics**										
Age at delivery (years)										
< 25	1054	8.1	984	6.5	1039	6.5	1318	8.5	2206	14.5
25–29	3407	26.2	3860	25.3	4133	25.8	4339	28.0	4959	32.6
30–34	4654	35.8	5722	37.5	6013	37.5	5643	36.4	4983	32.8
≥ 35	3869	29.8	4689	30.7	4846	30.2	4184	27.0	3069	20.2
**Smoking habits**										
Never smoked	7661	59.1	9633	63.2	10196	63.7	9198	59.5	8006	52.7
Ex-smokers who quit before pregnancy	2908	22.4	3555	23.3	3781	23.6	3825	24.7	3546	23.3
Smokers during early pregnancy	2392	18.5	2051	13.5	2043	12.8	2443	15.8	3645	24.0
**Alcohol consumption**										
Never drank	5002	38.5	5349	35.1	5329	33.3	5111	33.0	5027	33.1
Ex-drinkers who quit before pregnancy	2134	16.4	2845	18.7	3004	18.7	2923	18.9	2766	18.2
Drinkers during early pregnancy	5846	45.0	7059	46.3	7694	48.0	7446	48.1	7418	48.8
**Pre-pregnancy body mass index (kg/m**^**2**^**)**										
< 18.5	2191	16.9	2575	16.9	2469	15.4	2405	15.5	2400	15.8
18.5–24.9	9541	73.5	11209	73.5	12037	75.1	11534	74.5	11154	73.3
≥ 25.0	1252	9.6	1464	9.6	1513	9.5	1539	9.9	1660	10.9
**Parity**										
0	6065	46.8	6530	43.0	6900	43.2	6227	40.4	7548	49.8
≥ 1	6885	53.2	8673	57.1	9,077	56.8	9,197	59.6	7,625	50.3
**Infertility treatment**										
No	12,098	93.2	14,059	92.2	14,771	92.2	14,475	93.5	14,468	95.1
Ovulation stimulation/artificial insemination using husband’s sperms	471	3.6	635	4.2	648	4.0	574	3.7	467	3.1
Assisted reproductive technology	415	3.2	556	3.7	605	3.8	429	2.8	277	1.8
**Current history**										
Hypertensive disorders during pregnancy	421	3.2	362	2.4	415	2.6	379	2.5	501	3.3
Diabetes or gestational diabetes	379	2.9	451	3.0	499	3.1	477	3.1	447	2.9
**Type of delivery**										
Vaginal	10,623	81.9	12,602	82.8	13,285	83.1	12,765	82.6	12,648	83.3
Caesarean	2,345	18.1	2,617	17.2	2,710	16.9	2,685	17.4	2,536	16.7
**Gestational age (weeks)**										
Early term (37–38)	4,249	32.7	4,967	32.6	5,231	32.6	5,218	33.7	4,820	31.7
Full term (39–41)	8,735	67.3	10,288	67.4	10,800	67.4	10,266	66.3	10,397	68.3
**K6 Scale at 1 year after delivery**										
0–4	10,228	78.9	12,022	78.9	12,597	78.7	11,973	77.4	11,579	76.2
≥ 5 (psychological distress)	2728	21.1	3212	21.1	3413	21.3	3491	22.6	3612	23.8
**Educational background (years)**										
< 10	464	3.6	455	3.0	540	3.4	635	4.1	917	6.1
10–12	4500	34.9	4271	28.2	4210	26.5	4445	28.9	5213	34.6
13–16	7,801	60.5	10,148	67.0	10,784	67.9	9,985	65.0	8,825	58.5
≥ 17	125	1.0	271	1.8	355	2.2	292	1.9	123	0.8
**Household income (million Japanese yen/year)**										
< 2	645	5.4	595	4.2	597	4.0	680	4.7	1,086	7.8
2 to < 4	3995	33.4	4591	32.0	4687	31.2	4932	34.1	5508	39.4
4 to < 6	4005	33.5	5003	34.9	5210	34.7	4910	34.0	4245	30.3
6 to < 8	2040	17.0	2409	16.8	2650	17.7	2352	16.3	1895	13.5
8 to < 10	827	6.9	1038	7.2	1105	7.4	945	6.5	819	5.9
≥ 10	457	3.8	710	5.0	761	5.1	645	4.5	440	3.1
**Infant characteristics**										
Birth weight										
Mean (SD) (g)	3054 (362)		3065 (360)		3065 (368)		3066 (364)		3056 (368)	
Small for gestational age	949	7.3	1,015	6.7	1,198	7.5	1,063	6.9	1,196	7.9
Infant sex										
Male	6,582	50.7	7,723	50.6	8,226	51.3	7,888	50.9	7,773	51.1
Female	6,402	49.3	7,532	49.4	7,805	48.7	7,596	49.1	7,444	48.9
**Doctor diagnosis at 1-year-old**										
Asthma	305	2.4	325	2.1	351	2.2	360	2.3	481	3.2
Atopic dermatitis	516	4.0	654	4.3	744	4.6	649	4.2	723	4.8
**Feeding status**										
Formula feeding	378	2.9	296	1.9	299	1.9	273	1.8	381	2.5
Partial breastfeeding	8639	66.5	9740	63.9	10,046	62.7	9635	62.2	10,050	66.0
Exclusive breastfeeding	3967	30.6	5219	34.2	5686	35.5	5576	36.0	4786	31.5

*Numbers in subgroups do not equal overall number because of missing data.

*PA* physical activity, *MET* metabolic equivalent of a task, *IQR* interquartile range, *K6 scale* the Kessler six-item psychological distress scale, *SD* standard deviation.

### Association of maternal PA before and during pregnancy with sleep problems in 1-year-old infants

Low levels of maternal PA before and during pregnancy were associated with an increased risk ratio for bedtime after 22:00 but were not associated with other sleep outcomes (Tables 2 and 3). The risk ratio of bedtime after 22:00 in the group with the highest levels of PA (Quartile 4) both before and during pregnancy was lower than the reference group (PA before pregnancy, RR = 0.91, 95% CI = 0.87–0.95; PA during pregnancy, RR = 0.88, 95% CI = 0.84–0.92).

**Table 2:** Association between physical activity before pregnancy and infant sleep and development, Japan Environment and Children’s Study (2011–2014).

	No. of participants	No. of outcome		Maternal age adjusted model			Multivariable model^a^		
			%	RR	95% CI		RR	95% CI	
**Sleeping problems**									
3 or more awakening times in a night									
PA = 0	12,895	280	2.2	0.95	0.81	1.11	0.98	0.84	1.15
Quartile 1	15,158	350	2.3	Ref			Ref		
Quartile 2	15,923	426	2.7	1.16	1.01	1.33	1.14	0.99	1.31
Quartile 3	15,388	391	2.5	1.12	0.97	1.29	1.10	0.95	1.27
Quartile 4	15,128	357	2.4	1.08	0.93	1.24	1.11	0.95	1.28
**1 or more awakening times and staying awake for more than 1 h**									
PA = 0	12,895	736	5.7	1.08	0.98	1.19	1.05	0.95	1.16
Quartile 1	15,158	801	5.3	Ref			Ref		
Quartile 2	15,923	931	5.9	1.11	1.01	1.21	1.11	1.01	1.21
Quartile 3	15,388	880	5.7	1.08	0.99	1.19	1.07	0.98	1.18
Quartile 4	15,128	901	6.0	1.12	1.02	1.23	1.07	0.98	1.18
**Sleep for less than 8 h during the night (20:00 to 07:59)**									
PA = 0	12,895	712	5.5	1.12	1.01	1.24	1.10	1.00	1.22
Quartile 1	15,158	747	4.9	Ref			Ref		
Quartile 2	15,923	850	5.3	1.08	0.99	1.19	1.08	0.98	1.19
Quartile 3	15,388	799	5.2	1.06	0.96	1.16	1.06	0.96	1.17
Quartile 4	15,128	782	5.2	1.05	0.95	1.16	1.01	0.92	1.12
**Sleep at 22:00 or later**									
PA = 0	12,895	2723	21.1	1.01	0.97	1.06	0.99	0.94	1.03
Quartile 1	15,158	3140	20.7	Ref			Ref		
Quartile 2	15,923	3159	19.8	0.96	0.92	1.00	0.97	0.93	1.01
Quartile 3	15,388	2958	19.2	0.92	0.88	0.97	0.93	0.89	0.97
Quartile 4	15,128	2993	19.8	0.93	0.89	0.97	0.91	0.87	0.95
**Crying at night for 5 or more days in a week**									
PA = 0	12,975	904	7.0	0.96	0.88	1.04	0.99	0.91	1.07
Quartile 1	15,250	1111	7.3	Ref			Ref		
Quartile 2	16,031	1227	7.7	1.05	0.97	1.14	1.05	0.97	1.13
Quartile 3	15,474	1189	7.7	1.06	0.98	1.14	1.05	0.97	1.14
Quartile 4	15,211	1093	7.2	1.00	0.92	1.08	1.02	0.94	1.10
**The ages and stages questionnaire (ASQ)**									
Communication									
PA = 0	11,804	12	0.1	1.11	0.51	2.44	1.13	0.51	2.47
Quartile 1	13,953	13	0.1	Ref			Ref		
Quartile 2	14,687	17	0.1	1.24	0.60	2.56	1.24	0.60	2.56
Quartile 3	14,118	17	0.1	1.33	0.65	2.74	1.37	0.67	2.82
Quartile 4	13,916	8	0.1	0.69	0.28	1.66	0.69	0.29	1.68
**Gross motor skills**									
PA = 0	11,805	654	5.5	0.95	0.86	1.05	0.95	0.86	1.05
Quartile 1	13,953	820	5.9	Ref			Ref		
Quartile 2	14,685	825	5.6	0.96	0.87	1.05	0.97	0.88	1.06
Quartile 3	14,117	758	5.4	0.94	0.85	1.03	0.96	0.87	1.05
Quartile 4	13,920	682	4.9	0.90	0.82	1.00	0.93	0.84	1.02
**Fine motor skills**									
PA = 0	11,800	799	6.8	1.17	1.07	1.29	1.15	1.05	1.26
Quartile 1	13,949	814	5.8	Ref			Ref		
Quartile 2	14,679	830	5.7	0.97	0.88	1.07	0.97	0.88	1.07
Quartile 3	14,110	755	5.4	0.94	0.85	1.03	0.93	0.85	1.03
Quartile 4	13,913	620	4.5	0.82	0.74	0.91	0.81	0.73	0.90
**Problem-solving skills**									
PA = 0	11,784	760	6.5	1.23	1.12	1.36	1.20	1.09	1.33
Quartile 1	13,932	736	5.3	Ref			Ref		
Quartile 2	14,662	768	5.2	0.99	0.90	1.10	1.00	0.90	1.10
Quartile 3	14,102	629	4.5	0.86	0.78	0.96	0.87	0.79	0.97
Quartile 4	13,907	596	4.3	0.87	0.78	0.97	0.86	0.78	0.96
**Personal–social characteristics**									
PA = 0	11,771	171	1.5	1.27	1.02	1.57	1.29	1.04	1.60
Quartile 1	13,910	161	1.2	Ref			Ref		
Quartile 2	14,654	167	1.1	0.99	0.80	1.22	0.99	0.80	1.23
Quartile 3	14,084	163	1.2	1.03	0.83	1.27	1.04	0.84	1.30
Quartile 4	13,887	121	0.9	0.81	0.64	1.02	0.83	0.65	1.05
**Total (abnormal score for any 1 of the 5 domain)**									
PA = 0	11,810	1805	15.3	1.06	1.00	1.12	1.05	0.99	1.11
Quartile 1	13,958	2030	14.5	Ref			Ref		
Quartile 2	14,692	2013	13.7	0.94	0.89	1.00	0.95	0.89	1.00
Quartile 3	14,121	1817	12.9	0.90	0.85	0.96	0.91	0.86	0.97
Quartile 4	13,924	1582	11.4	0.84	0.79	0.89	0.84	0.79	0.90

*PA* physical activity, *CI* confidence interval, *RR* risk ratio, *Ref* reference.

^a^Adjusted for maternal age at delivery, smoking habits, alcohol consumption, pre-pregnancy body mass index, gestational age at birth, parity, infertility treatment, infant sex, type of delivery, psychological distress at 1 year after delivery, diagnosis of asthma and atopic dermatitis at 1-year-old, and feeding status.

**Table 3:** Association between physical activity during pregnancy and infant sleep and development, Japan Environment and Children’s Study (2011–2014).

	No. of participants	No. of outcome		Maternal age adjusted model			Multivariable model^a^		
			%	RR	95% CI		RR	95% CI	
**Sleeping problems**									
3 or more awakening times in a night									
PA = 0	16,611	365	2.2	0.93	0.80	1.08	0.95	0.82	1.10
Quartile 1	13,885	327	2.4	Ref			Ref		
Quartile 2	13,107	305	2.3	0.99	0.85	1.16	0.97	0.83	1.13
Quartile 3	14,653	403	2.8	1.18	1.02	1.36	1.16	1.00	1.34
Quartile 4	13,991	345	2.5	1.07	0.93	1.25	1.09	0.94	1.26
**1 or more awakening times and stayed awake for more than 1 h**									
PA = 0	16,611	958	5.8	0.99	0.90	1.08	1.00	0.91	1.09
Quartile 1	13,885	811	5.8	Ref			Ref		
Quartile 2	13,107	725	5.5	0.95	0.86	1.04	0.95	0.86	1.04
Quartile 3	14,653	823	5.6	0.96	0.88	1.06	0.96	0.88	1.06
Quartile 4	13,991	824	5.9	1.01	0.92	1.11	1.01	0.92	1.11
**Sleep for less than 8 h during the night (20:00 to 7:59)**									
PA = 0	16,611	860	5.2	0.98	0.89	1.08	0.99	0.90	1.09
Quartile 1	13,885	732	5.3	Ref			Ref		
Quartile 2	13,107	693	5.3	1.01	0.91	1.11	1.00	0.90	1.10
Quartile 3	14,653	770	5.3	1.00	0.91	1.11	1.00	0.91	1.10
Quartile 4	13,991	731	5.2	1.00	0.91	1.11	1.01	0.91	1.11
**Sleep at 22:00 or later**									
PA = 0	16,611	3367	20.3	0.96	0.92	1.00	0.95	0.91	0.99
Quartile 1	13,885	2945	21.2	Ref			Ref		
Quartile 2	13,107	2667	20.4	0.96	0.92	1.01	0.96	0.91	1.00
Quartile 3	14,653	2976	20.3	0.96	0.91	1.00	0.96	0.91	1.00
Quartile 4	13,991	2626	18.8	0.88	0.84	0.92	0.88	0.84	0.92
**Crying at night for 5 or more days in a week**									
PA = 0	16,721	1197	7.2	0.96	0.88	1.03	0.98	0.90	1.06
Quartile 1	13,967	1046	7.5	Ref			Ref		
Quartile 2	13,192	1008	7.6	1.02	0.94	1.11	1.01	0.93	1.10
Quartile 3	14,731	1096	7.4	0.99	0.92	1.08	0.99	0.91	1.07
Quartile 4	14,058	1015	7.2	0.97	0.89	1.05	0.99	0.91	1.07
**The ages and stages questionnaire (ASQ)**									
Communication									
PA = 0	15,207	15	0.1	0.74	0.37	1.48	0.76	0.38	1.52
Quartile 1	12,793	17	0.1	Ref			Ref		
Quartile 2	12,050	10	0.1	0.63	0.29	1.38	0.62	0.28	1.36
Quartile 3	13,536	16	0.1	0.91	0.46	1.80	0.90	0.46	1.78
Quartile 4	12,860	7	0.1	0.43	0.18	1.04	0.45	0.18	1.08
**Gross motor skills**									
PA = 0	15,212	902	5.9	1.04	0.95	1.14	1.05	0.95	1.15
Quartile 1	12,791	724	5.7	Ref			Ref		
Quartile 2	12,050	667	5.5	0.99	0.89	1.09	0.98	0.89	1.09
Quartile 3	13,533	700	5.2	0.93	0.84	1.03	0.94	0.85	1.04
Quartile 4	12,861	643	5.0	0.93	0.83	1.03	0.95	0.86	1.06
**Fine motor skills**									
PA = 0	15,200	1022	6.7	1.16	1.06	1.27	1.14	1.04	1.25
Quartile 1	12,790	733	5.7	Ref			Ref		
Quartile 2	12,043	635	5.3	0.92	0.83	1.03	0.93	0.84	1.03
Quartile 3	13,533	694	5.1	0.91	0.82	1.01	0.92	0.83	1.01
Quartile 4	12,854	622	4.8	0.88	0.79	0.98	0.87	0.78	0.97
**Problem-solving**									
PA = 0	15,182	922	6.1	1.15	1.05	1.27	1.14	1.03	1.25
Quartile 1	12,774	666	5.2	Ref			Ref		
Quartile 2	12,033	622	5.2	1.00	0.90	1.11	0.99	0.89	1.10
Quartile 3	13,521	648	4.8	0.94	0.84	1.04	0.94	0.85	1.04
Quartile 4	12,849	545	4.2	0.85	0.76	0.95	0.86	0.77	0.96
Personal–social characteristics									
PA = 0	15,168	213	1.4	1.29	1.04	1.60	1.27	1.03	1.58
Quartile 1	12,764	138	1.1	Ref			Ref		
Quartile 2	12,014	131	1.1	1.01	0.80	1.29	1.03	0.81	1.31
Quartile 3	13,509	147	1.1	1.02	0.81	1.29	1.05	0.84	1.33
Quartile 4	12,830	132	1.0	0.99	0.78	1.25	0.98	0.77	1.25
**Total (abnormal score for any 1 of the 5 domain)**									
PA = 0	15,214	2352	15.5	1.10	1.04	1.16	1.09	1.03	1.16
Quartile 1	12,798	1789	14.0	Ref			Ref		
Quartile 2	12,055	1601	13.3	0.96	0.90	1.02	0.96	0.90	1.02
Quartile 3	13,540	1738	12.8	0.93	0.88	0.99	0.95	0.89	1.01
Quartile 4	12,864	1517	11.8	0.88	0.82	0.94	0.89	0.84	0.95

*PA* physical activity, *CI* confidence interval, *RR* risk ratio, *Ref* reference.

^a^Adjusted for maternal age at delivery, smoking habits, alcohol consumption, pre-pregnancy body mass index, gestational age at birth, parity, infertility treatment, infant sex, type of delivery, psychological distress at 1 year after delivery, doctor diagnosis of asthma and atopic dermatitis at 1-year-old, and feeding status.

### Association of maternal PA before and during pregnancy with development in 1-year-old infants

Low levels of maternal PA both before and during pregnancy were associated with an increased risk of abnormal ASQ scores. Compared to the reference PA group before pregnancy, the group with the lowest levels of PA (PA = 0) had higher risk ratios of abnormal scores in the following domains of the ASQ: fine motor skills (RR = 1.15, 95% CI = 1.05–1.26), problem-solving (RR = 1.20, 95% CI = 1.09–1.33), and personal–social skills (RR = 1.29, 95% CI = 1.04–1.60) (Table 2).

Correspondingly, the risk ratios of abnormal scores for different domains of the ASQ were lower in the group with highest levels of PA (quartile 4) before pregnancy: fine motor skills (RR = 0.81, 95% CI = 0.73–0.90), problem-solving (RR = 0.86, 95% CI = 0.78–0.96), and overall skills (RR = 0.84, 95% CI = 0.79–0.90).

Similar associations were found in the analysis of PA during pregnancy (Table 3). Compared to the reference group for PA during pregnancy, the group with the lowest PA levels (PA = 0) showed higher risk ratios of abnormal scores in the following ASQ domains: fine motor skills (RR = 1.14, 95% CI = 1.04–1.25), problem-solving (RR = 1.14, 95% CI = 1.03–1.25), personal–social skills (RR = 1.27, 95% CI = 1.03–1.58), and overall skills (RR = 1.09, 95% CI = 1.03–1.16).

Conversely, in the group with the highest PA levels during pregnancy (quartile 4), the risk ratios of abnormal ASQ scores were lower in the following domains: fine motor skills (RR = 0.87, 95% CI = 0.78–0.97), problem-solving (RR = 0.86, 95% CI = 0.77–0.96), and overall skills (RR = 0.89, 95% CI = 0.84–0.95).

In other domains of the ASQ, including communication and gross motor skills, maternal PA both before and during pregnancy was not associated with significant risk ratios of abnormal scores.

The association between the risk ratios of abnormal ASQ scores and maternal PA levels, both before and during pregnancy, did not change in the subgroup analysis that excluded women with HDP and GDM (Table S2).

## Discussion

In the present study, lower maternal PA levels before and during pregnancy increased the risk ratios of abnormal scores on the infants’ ASQ at 1 year of age. Higher maternal PA levels were associated with lower risk ratios of abnormal ASQ scores. Similarly, maternal PA levels before and during pregnancy were inversely associated with infant late bedtime at or after 22:00, as shown by the risk ratio. However, other sleep outcomes were not associated with maternal PA levels. This is the first study to show that maternal PA levels before pregnancy influence measures of development and concur with previous findings associated with PA during pregnancy. Higher PA levels during preconception may decrease the risk of developmental delay.

Regarding infant sleep problems, lower maternal PA levels before and during pregnancy were associated exclusively with late bedtime in 1-year-old infants. In this study, the proportion of infants who fell asleep after 22:00 was approximately 20%, which is a larger percentage than other sleep outcomes. Thus, a small but significant difference in late bedtime was detected. A high level of maternal activity during pregnancy has been reported to improve maternal sleep^15,16^. The sleep cycle develops from the fetal period^17^, and the association between maternal sleep during pregnancy and infants’ sleep has also been reported^18,19^. Therefore, maternal PA during pregnancy may affect infants’ sleep through maternal sleep. No other studies have addressed the direct association between maternal PA and infant sleep patterns. Further investigations are required to evaluate these associations.

This is the first study to show that maternal PA before pregnancy may influence infant developmental outcomes. Regarding PA during pregnancy, there have been many previous studies that reported an association between PA during pregnancy and language development^20–22^. The association between PA during pregnancy, motor function, and social skills remains inconclusive^5,6^. A recent RCT study reported a positive association between maternal exercise and infant neuromotor outcomes at 1- month-old^23^. Although there was a significant association between maternal PA and child development, it was also reported that the association became insignificant as the child matured^21,24^. Future studies should investigate the association between maternal PA before and during pregnancy and development in older children.

There are several hypotheses on how maternal PA before and during pregnancy affects infant neurodevelopment. The first hypothesis involves maternal inflammation, which affects fetal neurodevelopment in utero and may cause developmental disorders^25^. One study reported that exercise intervention in pregnant women reduced inflammatory cytokines^26^. Therefore, high maternal PA levels may protect fetal neurodevelopment from inflammation.

The second hypothesis is that maternal PA directly affects neurodevelopment in infants. In an experiment with mice and rats, exercise during pregnancy improved neurogenesis in the hippocampus, memory, and learning outcomes^27–31^. From this study, it can be inferred that for humans, PA during pregnancy may have a beneficial influence on fetal neurodevelopment.

The third hypothesis is that maternal activity may stimulate fetal sensory systems, such as vestibular function. A study of preterm infants reported that auditory, tactile, visual, and vestibular interventions increased nipple feeding and decreased the length of infant hospitalization^32^. As fetal vestibular function develops from early pregnancy^33,34^, maternal PA may stimulate the fetal vestibular system to positively affect neurodevelopment.

Perinatal complications, such as GDM, HDP, and perinatal depression, have negative effects on child neurodevelopment^1,2,35,36^. These complications are known risk factors of developmental disorders^11^. However, in our subgroup analysis, which excluded cases of HDP and GDM, an association between abnormalities in ASQ scores and maternal PA before and during pregnancy was found. This finding implies that HDP and GDM may not be the only complications associated with maternal PA before and during pregnancy.

This study has several limitations. First, this was an observational study, so there could be unmeasured confounding factors, such as parental life rhythm or sleep cycle. Second, maternal PA and infant outcomes (infant sleep problems and ASQ scores) were evaluated using a self-reported questionnaire, so there could be some bias. In particular, maternal PA before pregnancy was reported at recruitment in the first trimester of pregnancy. On the other hand, the strength of the present study was that it was based on national data. Additionally, this was the first study to focus on the association between maternal PA before pregnancy and infant development.

In conclusion, lower maternal PA before or during pregnancy was associated with negative effects on infant development and increased risk of late bedtimes in 1-year-old infants. In contrast, higher maternal PA before or during pregnancy may have positive effects on infant development and decrease the risk of late bedtimes in 1-year-old infants.

## Methods

### Research ethics

The study protocol was approved by the Ministry of the Environment’s Institutional Review Board on Epidemiological Studies (No. 100406001) and by the Ethics Committee of all participating institutions: the National Institute for Environmental Studies that leads the Japan Environment and Children’s Study (JECS), the National Center for Child Health and Development, Hokkaido University, Sapporo Medical University, Asahikawa Medical College, Japanese Red Cross Hokkaido College of Nursing, Tohoku University, Fukushima Medical University, Chiba University, Yokohama City University, University of Yamanashi, Shinshu University, University of Toyama, Nagoya City University, Kyoto University, Doshisha University, Osaka University, Osaka Medical Center and Research Institute for Maternal and Child Health, Hyogo College of Medicine, Tottori University, Kochi University, University of Occupational and Environmental Health, Kyushu University, Kumamoto University, University of Miyazaki, and University of Ryukyu. Written informed consent, which also included a follow-up study of children after birth, was obtained from all participants. All methods were performed in accordance with approved guidelines.

### Study participants

The data used in this study were obtained from the JECS, an ongoing large-scale cohort study. The JECS was designed to follow children from the prenatal period to the age of 13 years. The detailed protocol of the study and the baseline profile of participants in the JECS have been previously reported previously^37,38^. The participants answered a questionnaire about lifestyle and behavior twice during pregnancy. The questionnaire completed at recruitment was referred to as M-T1, and the questionnaire completed later during mid- and late pregnancy was M-T2. The mean gestational weeks (SD) at the time of responding to M-T1 and M-T2 were 16.4 (8.0) and 27.9 (6.5) weeks, respectively. Participants also answered a questionnaire about their offspring one year after delivery (C-1y).

**Figure 1:**
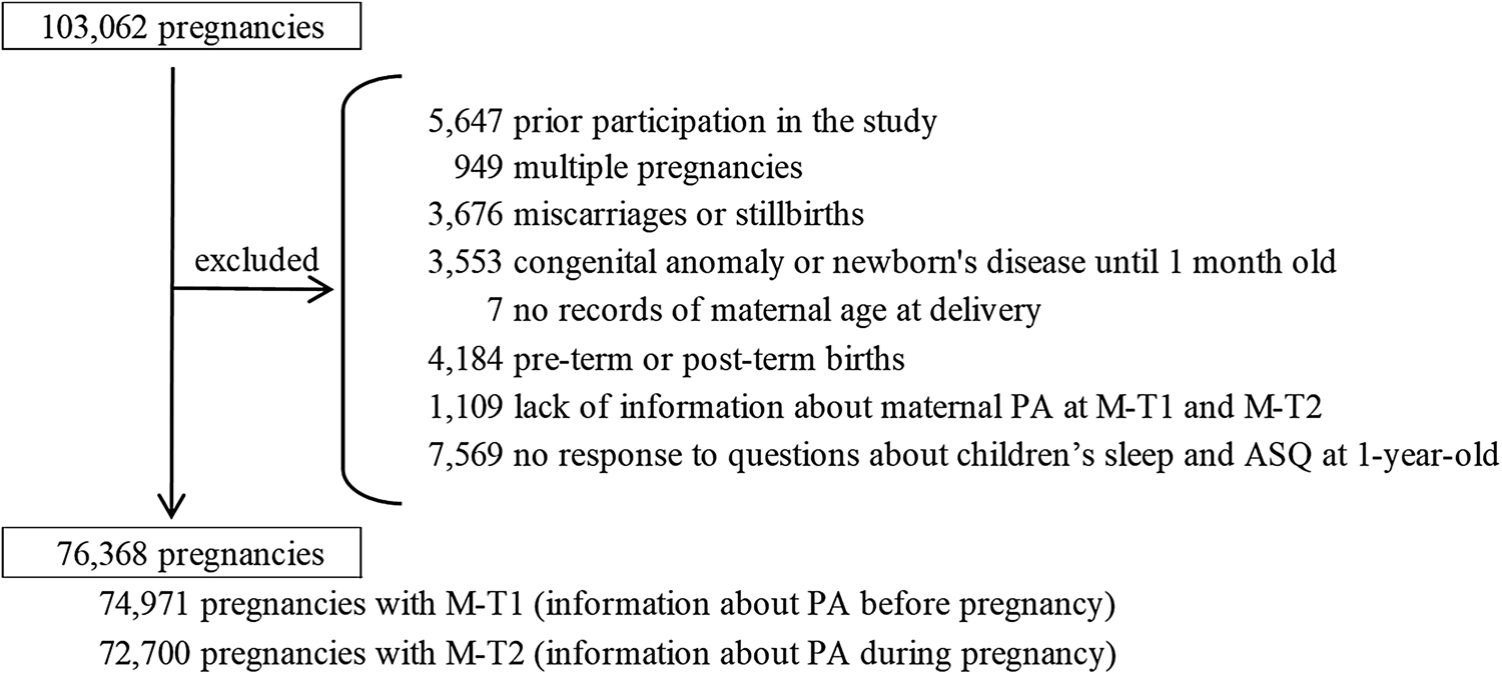
Population flowchart. PA, physical activity; M-T1, questionnaire administered at recruitment; M-T2, questionnaire administered during mid- and late pregnancy; ASQ, Ages and Stages Questionnaire.

Between 2011 and 2014, 103,062 pregnant women were recruited from 15 regions throughout Japan (Fig. 1). Of these, we excluded 26,694 pregnancies due to the following reasons: previous participation in the study (n = 5647), multiple fetuses (n = 949), miscarriage or stillbirth (n = 3,676), congenital anomaly or disease at 1 month of age (n = 3553), missing information on maternal age at delivery (n = 7), delivery before 37 weeks or after 42 weeks of gestation (n = 4184), lack of information about maternal PA in the M-T1 and M-T2 (n = 1109), and no response to questions about children’s sleep and development at C-1y (n = 7569). The remaining 76,368 participants (74,971 with M-T1 data and 72,700 with M-T2 data) were included in the analysis.

### Exposure: maternal PA

We used the Japanese short version of the International Physical Activity Questionnaire (IPAQ) to evaluate maternal PA, for which test–retest reliability and criterion validity were reported elsewhere^39,40^. Participants reported their mean PA per week before pregnancy on the M-T1 questionnaire based on recall and their mean PA per week during pregnancy on the M-T2 questionnaire. We calculated PA in terms of metabolic equivalent of a task (MET), measured as the number of minutes per week (METs-min/week)^39^. PA, as defined in the IPAQ, includes all activities of daily life, such as work, housework, and leisure activities.

We divided the participants into five groups based on their level of PA before pregnancy. We also divided the participants into five groups based on their levels of PA during pregnancy. In each of the five groups, the “PA = 0” group consisted of participants whose PA was 0. The other participants were divided into four groups using PA quartile points. The groups were labeled Quartiles 1–4 in ascending order of PA. Quartile 1 referred to the group with the lowest PA levels among the four groups. Quartile 4 referred to the group with the highest PA levels. To visualize the effects when the amount of activity was very low, we defined the Quartile 1 groups as the reference groups for the purpose of statistical analysis instead of the PA = 0 groups.

### Outcome 1: infant sleeping problems

One year after delivery, information on infant sleep habits was collected via parent-reported questionnaires (C-1y). The participants answered questions regarding their infant’s sleep time in the previous 24 h, in 30-min increments. They were also asked whether their children cried at night, and if so, the crying frequency (“rarely,” “1–3 times in a month,” “1–2 times in a week,” “3–4 times in a week,” “5 times in a week or more”) was reported. In this analysis, we focused on five points. First, we determined the number of nocturnal awakenings from maternal responses to infants’ sleeping periods. We defined ≥ 3 awakenings as too many because a previous study reported that the upper limit of the number of awakenings during the night was 2.5 for 1-year-old infants^41^. Second, we determined whether the infants awoke more than once and whether they stayed awake for more than 1 h during the night. If so, these were defined as unusual. Third, we analyzed the duration of nighttime sleep (20:00–07:59). We regarded less than 8 h of sleep as too short because past research reported that the mean duration of sleep for this age group was 8.3 h^41^. Fourth, we determined the infants’ bedtime. In this study, about 65% of 1-year-old infants slept later than 21:00, and about 20% slept later than 22:00. Therefore, we defined bedtime after 22:00 as too late. Fifth, we obtained information about crying at night in the past month. If the mother answered that her infant cried during the night, and the frequency of crying at night was more than five times per week, we defined the case as “crying at night”.

### Outcome 2: infant development

We used the Japanese version of the Ages and Stages Questionnaire (ASQ), third edition, to evaluate infant development. The C-1y questionnaire included ASQ. ASQ captures developmental delay in five domains: communication, gross motor skills, fine motor skills, problem-solving, and personal–social characteristics. The answer to each question is one of the following: “yes,” “sometimes,” or “not yet.” The scores were 10, 5, and 0 points, respectively. Each ASQ domain was composed of six questions, and the total score ranged from 0 to 60. The cut-off point for each domain in the Japanese version was 2SD below the mean, and all the cut-off points were determined by age groups in a previous study^42^. The cut-off points at 1-year-old are as follows: communication, 4.53; gross motor skill, 9.43; fine motor skill, 25.47; problem solving, 15.37; and personal-social characteristics, 4.95. The outcomes were defined as whether the score was less than the cut-off point for each ASQ domain and whether the score was less than the cut-off point of any one of the five ASQ domains.

### Covariates

Information on maternal age at delivery, pre-pregnancy body mass index (BMI), parity, gestational age at birth, infertility treatment, type of delivery, current history of hypertensive disorders of pregnancy and diabetes or gestational diabetes, infant birth weight, and infant sex were collected from medical records. Information about smoking habits, alcohol consumption, educational background, household income, maternal psychological distress at 1 year after delivery, doctor diagnosis of asthma and atopic dermatitis in children up to 1 year of age, and feeding status were collected via self-administered questionnaires. Maternal depression has been reported to affect infant development^43^. In the present study, we did not know whether the participants had a mental illness after delivery. Thus, maternal psychological distress was assessed using the Kessler 6^44,45^ questionnaire at C-1y. In concordance with previous studies, participants with a score of five or more were categorized as having distress^46^.

### Statistical analyses

We used a log-binominal regression model to explore the association of maternal PA with each outcome and to estimate the risk ratio (RR) of each outcome and the 95% confidence intervals (CIs). We initially adjusted for maternal age at delivery and then further adjusted for smoking habits (never smokers, ex-smokers who quit before pregnancy, smokers during early pregnancy), alcohol consumption (never drinkers, ex-drinkers who quit before pregnancy, drinkers during early pregnancy), pre-pregnancy BMI (< 18.5, 18.5–24.9, ≥ 25.0 kg/m^2^), parity (0, ≥ 1), infertility treatment (no ovulation stimulation/artificial insemination by sperm from husband, assisted reproductive technology), type of delivery (vaginal or cesarean section), gestational age at birth (37–38, 39–41 weeks), infant sex (boys, girls), psychological distress at 1 year after delivery (yes, no), doctor diagnosis of asthma and atopic dermatitis at 1 year of age, and feeding (breast milk, formula, both). The covariates to be added to the multivariate model were determined by referring to the previous literature as potential risk factors for developmental disorders^11,47^. Due to the large sample size in this study, we used the risk factors contained in the dataset in the multivariate model as covariates whenever possible. However, since there were many missing data on household income and educational background, we excluded them from the covariates of the multivariate model after confirming that the results did not change significantly even if they were included in the model. We did not complete the missing data. Thus, the multivariate analysis was limited to those participants that had all the covariate data.

We also performed a subgroup analysis excluding women with HDP and GDM to investigate the influence of these factors on infant development.

In this study, we used a fixed dataset “jecs-an-20180131,” which was released in March 2018. Stata version 15 (StataCorp LP, College Station, TX, USA) was used for all statistical analyses.

The statistical analyses of this study were conducted in a manner that was similar to that of our previous study^48^.

## Supplementary Information

Supplementary Information.
