# Supplementary material for: Influence of physical activity before and during pregnancy on infant’s sleep and neurodevelopment at 1-year-old
Source: Sci Rep. 2021 Apr 14;11:8099. doi: 10.1038/s41598-021-87612-1 (PMC8046980; doi:10.1038/s41598-021-87612-1)
Supplement: Supplementary file 1 — Supplementary Information. [file 41598_2021_87612_MOESM1_ESM.docx]

**Supplemental table 1-2**

**Influence of physical activity before and during pregnancy on infant’s sleep and neurodevelopment at 1-year-old**

Kazushige Nakahara^1**^, Takehiro Michikawa^2**^, Seiichi Morokuma*^3, 4^,

Masanobu Ogawa^4^, Kiyoko Kato^1, 4^, Masafumi Sanefuji^4, 5^,

Eiji Shibata^6, 7^, Mayumi Tsuji^6, 8^, Masayuki Shimono^6, 9^, Toshihiro Kawamoto^6^, Shouichi Ohga^5^, Koichi Kusuhara^6, 9^, and the Japan Environment and Children’s Study Group^***^

| **Supplemental Table 1**. Baseline characteristics of the study population categorized by physical activity during pregnancy | | | | | | | | | | |  |  |
| --- | --- | --- | --- | --- | --- | --- | --- | --- | --- | --- | --- | --- |
|  |  |  |  |  |  |  |  |  |  |  |  |  |
|  |  |  | Physical activity during pregnancy | | | | | | | | | |
|  |  |  | PA=0 | | Quartile 1 | | Quartile 2 | | Quartile 3 | | Quartile 4 | |
|  |  |  | n* | % | n* | % | n* | % | n* | % | n* | % |
| Physical activity, median (IQR), METSs・min / week | | | 0 | | 196 (119-280) | | 497 (399-595) | | 1190 (987-1470) | | 4774 (2968-7917) | |
|  |  |  |  |  |  |  |  |  |  |  |  |  |
| Maternal characteristics | | |  |  |  |  |  |  |  |  |  |  |
|  | Age at delivery (years) | |  |  |  |  |  |  |  |  |  |  |
|  |  | < 25 | 1,269 | 7.6 | 1,152 | 8.2 | 1,067 | 8.1 | 1,385 | 9.4 | 1,593 | 11.3 |
|  |  | 25-29 | 4,171 | 24.9 | 3,556 | 25.5 | 3,627 | 27.5 | 4,218 | 28.6 | 4,482 | 31.9 |
|  |  | 30-34 | 6,063 | 36.2 | 5,196 | 37.2 | 4,742 | 35.9 | 5,329 | 36.2 | 4,875 | 34.7 |
|  |  | ≥ 35 | 5,226 | 31.2 | 4,070 | 29.1 | 3,759 | 28.5 | 3,804 | 25.8 | 3,116 | 22.2 |
|  | Smoking habits | |  |  |  |  |  |  |  |  |  |  |
|  |  | Never smoked | 10,074 | 60.3 | 8,574 | 61.4 | 8,094 | 61.4 | 8,929 | 60.7 | 7,646 | 54.4 |
|  |  | Ex-smokers who quit before pregnancy | 3,772 | 22.6 | 3,309 | 23.7 | 3,106 | 23.6 | 3,458 | 23.5 | 3,462 | 24.7 |
|  |  | Smokers during early pregnancy | 2,866 | 17.2 | 2,075 | 14.9 | 1,983 | 15.0 | 2,334 | 15.9 | 2,937 | 20.9 |
|  | Alcohol consumption | |  |  |  |  |  |  |  |  |  |  |
|  |  | Never drank | 6,135 | 36.7 | 4,804 | 34.4 | 4,466 | 33.9 | 4,872 | 33.1 | 4,736 | 33.7 |
|  |  | Ex-drinkers who quit before pregnancy | 2,821 | 16.9 | 2,510 | 18.0 | 2,372 | 18.0 | 2,734 | 18.6 | 2,707 | 19.3 |
|  |  | Drinkers during early pregnancy | 7,772 | 46.5 | 6,657 | 47.7 | 6,355 | 48.2 | 7,128 | 48.4 | 6,616 | 47.1 |
|  | Pre-pregnancy body mass index (kg/m^2^) | |  |  |  |  |  |  |  |  |  |  |
|  |  | <18.5 | 2,755 | 16.5 | 2,275 | 16.3 | 2,123 | 16.1 | 2,382 | 16.2 | 2,180 | 15.5 |
|  |  | 18.5–24.9 | 12,318 | 73.7 | 10,399 | 74.5 | 9,776 | 74.1 | 10,934 | 74.3 | 10,332 | 73.5 |
|  |  | ≥25.0 | 1,650 | 9.9 | 1,289 | 9.2 | 1,289 | 9.8 | 1,410 | 9.6 | 1,550 | 11.0 |
|  | Parity | |  |  |  |  |  |  |  |  |  |  |
|  |  | 0 | 7,087 | 42.5 | 6,347 | 45.6 | 6,258 | 47.6 | 6,821 | 46.4 | 5,822 | 41.5 |
|  |  | ≥ 1 | 9,585 | 57.5 | 7,576 | 54.4 | 6,882 | 52.4 | 7,869 | 53.6 | 8,198 | 58.5 |
|  | Infertility treatment | |  |  |  |  |  |  |  |  |  |  |
|  |  | No | 15,583 | 93.2 | 12,928 | 92.6 | 12,158 | 92.2 | 13,731 | 93.2 | 13,351 | 95.0 |
|  |  | Ovulation stimulation/artificial insemination using husband’s sperms | 606 | 3.6 | 562 | 4.0 | 546 | 4.1 | 555 | 3.8 | 447 | 3.2 |
|  |  | Assisted reproductive technology | 529 | 3.2 | 479 | 3.4 | 483 | 3.7 | 440 | 3.0 | 262 | 1.9 |
|  | Current history | |  |  |  |  |  |  |  |  |  |  |
|  |  | Hypertensive disorders of pregnancy | 507 | 3.0 | 394 | 2.8 | 353 | 2.7 | 395 | 2.7 | 380 | 2.7 |
|  |  | Diabetes or gestational diabetes | 455 | 2.7 | 446 | 3.2 | 438 | 3.3 | 444 | 3.0 | 382 | 2.7 |
|  | Type of delivery | |  |  |  |  |  |  |  |  |  |  |
|  |  | Vaginal | 13,644 | 81.7 | 11,499 | 82.5 | 10,887 | 82.6 | 12,347 | 84.0 | 11,682 | 83.2 |
|  |  | Caesarean | 3,056 | 18.3 | 2,447 | 17.6 | 2,288 | 17.4 | 2,346 | 16.0 | 2,356 | 16.8 |
|  | Gestational age (weeks) | |  |  |  |  |  |  |  |  |  |  |
|  |  | Early term (37-38) | 5,728 | 34.2 | 4,578 | 32.8 | 4,167 | 31.6 | 4,623 | 31.4 | 4,625 | 32.9 |
|  |  | Full term (39-41) | 11,001 | 65.8 | 9,396 | 67.2 | 9,028 | 68.4 | 10,113 | 68.6 | 9,441 | 67.1 |
|  | K6 scale at 1 year after delivery | |  |  |  |  |  |  |  |  |  |  |
|  |  | 0-4 | 13,328 | 79.8 | 10,892 | 78.0 | 10,178 | 77.3 | 11,413 | 77.5 | 10,808 | 77.0 |
|  |  | >=5 (Psychological distress) | 3,378 | 20.2 | 3,065 | 22.0 | 2,988 | 22.7 | 3,306 | 22.5 | 3,233 | 23.0 |
|  | Educational background (years) | |  |  |  |  |  |  |  |  |  |  |
|  |  | < 10 | 588 | 3.5 | 516 | 3.7 | 509 | 3.9 | 653 | 4.5 | 701 | 5.0 |
|  |  | 10-12 | 5,457 | 32.7 | 4,102 | 29.5 | 3,897 | 29.6 | 4,231 | 28.8 | 4,465 | 31.9 |
|  |  | 13-16 | 10,430 | 62.5 | 9,069 | 65.1 | 8,497 | 64.6 | 9,513 | 64.8 | 8,696 | 62.1 |
|  |  | ≥ 17 | 202 | 1.2 | 241 | 1.7 | 255 | 1.9 | 290 | 2.0 | 144 | 1.0 |
|  | Household income (million Japanese-yen/year) | |  |  |  |  |  |  |  |  |  |  |
|  |  | < 2 | 794 | 5.1 | 638 | 4.9 | 558 | 4.5 | 722 | 5.2 | 794 | 6.1 |
|  |  | 2 to < 4 | 4,959 | 31.8 | 4,444 | 34.1 | 4,221 | 34.1 | 4,770 | 34.5 | 4,652 | 35.5 |
|  |  | 4 to < 6 | 5,181 | 33.3 | 4,439 | 34.0 | 4,317 | 34.8 | 4,662 | 33.7 | 4,177 | 31.8 |
|  |  | 6 to < 8 | 2,789 | 17.9 | 2,085 | 16.0 | 1,964 | 15.8 | 2,191 | 15.8 | 2,068 | 15.8 |
|  |  | 8 to < 10 | 1,183 | 7.6 | 808 | 6.2 | 808 | 6.5 | 902 | 6.5 | 906 | 6.9 |
|  |  | ≥ 10 | 667 | 4.3 | 625 | 4.8 | 528 | 4.3 | 582 | 4.2 | 521 | 4.0 |
| Infant characteristics | | |  |  |  |  |  |  |  |  |  |  |
|  | Birth weight | |  |  |  |  |  |  |  |  |  |  |
|  |  | Mean (SD) (g) | 3,057 (364) | | 3,061 (358) | | 3,065 (369) | | 3,065 (365) | | 3,061 (366) | |
|  |  | Small for gestational age | 1,185 | 7.1 | 948 | 6.8 | 992 | 7.6 | 1,061 | 7.2 | 1,049 | 7.5 |
|  | Infant sex | |  |  |  |  |  |  |  |  |  |  |
|  |  | Male | 8,503 | 50.8 | 7,072 | 50.6 | 6,734 | 51.0 | 7,573 | 51.4 | 7,139 | 50.8 |
|  |  | Female | 8,226 | 49.2 | 6,902 | 49.4 | 6,461 | 49.0 | 7,163 | 48.6 | 6,927 | 49.3 |
|  | Doctor-diagnosis at 1-year-old | |  |  |  |  |  |  |  |  |  |  |
|  |  | Asthma | 430 | 2.6 | 283 | 2.0 | 275 | 2.1 | 305 | 2.1 | 483 | 3.4 |
|  |  | Atopic dermatitis | 666 | 4.0 | 649 | 4.6 | 583 | 4.4 | 653 | 4.4 | 629 | 4.5 |
|  | Feeding status | |  |  |  |  |  |  |  |  |  |  |
|  |  | Formula feeding | 457 | 2.7 | 267 | 1.9 | 243 | 1.8 | 272 | 1.9 | 335 | 2.4 |
|  |  | Partial breastfeeding | 10,931 | 65.3 | 8,898 | 63.7 | 8,416 | 63.8 | 9,223 | 62.6 | 9,215 | 65.5 |
|  |  | Exclusive breastfeeding | 5,341 | 31.9 | 4,809 | 34.4 | 4,536 | 34.4 | 5,241 | 35.6 | 4,516 | 32.1 |
| *Numbers in subgroups do not equal overall number because of missing data. | | | | | |  |  |  |  |  |  |  |
| PA physical activity: MET metabolic equivalent of a task: IQR interquartile range: K6 scale: the Kessler Six-item Psychological Distress scale: SD standard deviation | | | | | | | | | | | | |

| **Supplemental** **Table 2.** Association between physical activity during preconception and offspring's development among women without history of gestational hypertensive disorders and/or diabetes or gestational diabetes, Japan Environment and Children's Study (2011-2014) | | | | | | | | | | | | | | | | |
| --- | --- | --- | --- | --- | --- | --- | --- | --- | --- | --- | --- | --- | --- | --- | --- | --- |
|  |  |  |  |  |  |  |  |  |  |  |  |  |  |  | |  |
|  |  |  | **Physical activity before pregnancy** | | | | | |  | **Physical activity during pregnancy** | | | | | | |
|  |  |  | **No. of participants** | **No. of outcome** | | **Multivariable model ^a^** | | |  | **No. of participants** | **No. of outcome** | | **Multivariable model ^a^** | | | |
|  |  |  |  |  | **%** | **RR** | **95% CI** | |  |  |  | **%** | **RR** | **95% CI** | | |
| The Ages and Stages Questionnaires (ASQ) | | | | |  |  |  |  |  |  |  |  |  |  | |  |
|  | **Communication** | |  |  |  |  |  |  |  |  |  |  |  |  | |  |
|  | PA=0 | | 11,100 | 10 | 0.1 | **1.12** | 0.47 | 2.64 |  | 14,363 | 14 | 0.1 | **0.92** | 0.43 | | 1.97 |
|  | Quartile 1 | | 13,232 | 11 | 0.1 | **Ref** |  |  |  | 12,045 | 13 | 0.1 | **Ref** |  | |  |
|  | Quartile 2 | | 13,877 | 17 | 0.1 | **1.48** | 0.69 | 3.15 |  | 11,348 | 10 | 0.1 | **0.81** | 0.36 | | 1.85 |
|  | Quartile 3 | | 13,372 | 17 | 0.1 | **1.62** | 0.76 | 3.46 |  | 12,796 | 15 | 0.1 | **1.10** | 0.52 | | 2.31 |
|  | Quartile 4 | | 13,083 | 7 | 0.1 | **0.72** | 0.28 | 1.87 |  | 12,187 | 7 | 0.1 | **0.58** | 0.23 | | 1.47 |
|  | **Gross motor skills** | | |  |  |  |  |  |  |  |  |  |  |  | |  |
|  | PA=0 | | 11,102 | 608 | 5.5 | **0.95** | 0.86 | 1.06 |  | 14,368 | 833 | 5.8 | **1.04** | 0.94 | | 1.14 |
|  | Quartile 1 | | 13,232 | 770 | 5.8 | **Ref** |  |  |  | 12,044 | 675 | 5.6 | **Ref** |  | |  |
|  | Quartile 2 | | 13,875 | 770 | 5.6 | **0.97** | 0.88 | 1.06 |  | 11,348 | 630 | 5.6 | **1.00** | 0.90 | | 1.11 |
|  | Quartile 3 | | 13,371 | 721 | 5.4 | **0.97** | 0.88 | 1.07 |  | 12,793 | 662 | 5.2 | **0.95** | 0.86 | | 1.06 |
|  | Quartile 4 | | 13,088 | 633 | 4.8 | **0.92** | 0.83 | 1.02 |  | 12,189 | 608 | 5.0 | **0.96** | 0.86 | | 1.07 |
|  | **Fine motor skills** | | |  |  |  |  |  |  |  |  |  |  |  | |  |
|  | PA=0 | | 11,096 | 742 | 6.7 | **1.14** | 1.03 | 1.26 |  | 14,358 | 946 | 6.6 | **1.13** | 1.03 | | 1.25 |
|  | Quartile 1 | | 13,229 | 768 | 5.8 | **Ref** |  |  |  | 12,043 | 682 | 5.7 | **Ref** |  | |  |
|  | Quartile 2 | | 13,870 | 780 | 5.6 | **0.97** | 0.88 | 1.07 |  | 11,341 | 594 | 5.2 | **0.94** | 0.84 | | 1.04 |
|  | Quartile 3 | | 13,365 | 712 | 5.3 | **0.93** | 0.84 | 1.03 |  | 12,793 | 664 | 5.2 | **0.94** | 0.85 | | 1.04 |
|  | Quartile 4 | | 13,080 | 579 | 4.4 | **0.81** | 0.73 | 0.90 |  | 12,181 | 585 | 4.8 | **0.87** | 0.78 | | 0.97 |
|  | **Problem-solving** | | |  |  |  |  |  |  |  |  |  |  |  | |  |
|  | PA=0 | | 11,081 | 703 | 6.3 | **1.21** | 1.09 | 1.34 |  | 14,343 | 852 | 5.9 | **1.15** | 1.04 | | 1.27 |
|  | Quartile 1 | | 13,216 | 684 | 5.2 | **Ref** |  |  |  | 12,028 | 608 | 5.1 | **Ref** |  | |  |
|  | Quartile 2 | | 13,855 | 708 | 5.1 | **0.99** | 0.89 | 1.10 |  | 11,331 | 573 | 5.1 | **1.00** | 0.89 | | 1.11 |
|  | Quartile 3 | | 13,359 | 591 | 4.4 | **0.88** | 0.79 | 0.99 |  | 12,784 | 613 | 4.8 | **0.97** | 0.87 | | 1.08 |
|  | Quartile 4 | | 13,074 | 549 | 4.2 | **0.87** | 0.77 | 0.97 |  | 12,177 | 513 | 4.2 | **0.88** | 0.78 | | 0.99 |
|  | **Personal-social characteristics** | | |  |  |  |  |  |  |  |  |  |  |  | |  |
|  | PA=0 | | 11,067 | 161 | 1.5 | **1.27** | 1.02 | 1.59 |  | 14,325 | 200 | 1.4 | **1.29** | 1.03 | | 1.61 |
|  | Quartile 1 | | 13,194 | 152 | 1.2 | **Ref** |  |  |  | 12,017 | 128 | 1.1 | **Ref** |  | |  |
|  | Quartile 2 | | 13,846 | 152 | 1.1 | **0.94** | 0.75 | 1.18 |  | 11,315 | 122 | 1.1 | **1.04** | 0.81 | | 1.33 |
|  | Quartile 3 | | 13,340 | 152 | 1.1 | **0.99** | 0.79 | 1.24 |  | 12,773 | 135 | 1.1 | **1.04** | 0.82 | | 1.32 |
|  | Quartile 4 | | 13,056 | 115 | 0.9 | **0.84** | 0.66 | 1.08 |  | 12,159 | 127 | 1.0 | **1.02** | 0.79 | | 1.30 |
|  | **Total (abnormal score for any 1 of 5 domains)** | | | | |  |  |  |  |  |  |  |  |  | |  |
|  | PA=0 | | 11,106 | 1,683 | 15.2 | **1.05** | 0.99 | 1.12 |  | 14,370 | 2,178 | 15.2 | **1.09** | 1.03 | | 1.16 |
|  | Quartile 1 | | 13,237 | 1,904 | 14.4 | **Ref** |  |  |  | 12,050 | 1,655 | 13.7 | **Ref** |  | |  |
|  | Quartile 2 | | 13,882 | 1,871 | 13.5 | **0.94** | 0.89 | 1.00 |  | 11,353 | 1,501 | 13.2 | **0.97** | 0.91 | | 1.04 |
|  | Quartile 3 | | 13,375 | 1,713 | 12.8 | **0.92** | 0.86 | 0.97 |  | 12,800 | 1,647 | 12.9 | **0.96** | 0.91 | | 1.03 |
|  | Quartile 4 | | 13,091 | 1,472 | 11.2 | **0.84** | 0.79 | 0.90 |  | 12,191 | 1,428 | 11.7 | **0.90** | 0.84 | | 0.96 |
| CI, confidence interval; RR, risk ratio; PA, physical activity; Ref, reference | | | | | | | | | | | | | | |  |  |
| ^a^ Adjusted for maternal age at delivery, smoking habit, alcohol consumption, pre-pregnancy body mass index, gestational age at birth, parity, infertility treatment, infant sex, type of delivery, psychological distress at 1 year after delivery, diagnosis of asthma and atopic dermatitis at 1-year-old, and feeding status. | | | | | | | | | | | | | | |  |  |
